# Physical, Mental, and Financial Stress Impacts of COVID-19 on Early Childhood Educators

**DOI:** 10.1007/s10643-021-01223-z

**Published:** 2021-06-28

**Authors:** Nancy L. Swigonski, Brandy James, Whitley Wynns, Kara Casavan

**Affiliations:** 1 Department of Pediatrics, Indiana University School of Medicine, 410 W. 10th St, Suite 2000, Indianapolis, IN 46202 USA; 2 Riley Children’s Health, 705 Riley Hospital Dr, Indianapolis, IN 46202 USA; 3 Department of Health Policy and Management, Fairbanks School of Public Health, Indiana University, Indianapolis, USA; 4 Department of Early Childhood Education, Indiana University School of Education, Indianapolis, USA

In the United States, more than two million early childhood educators care for approximately ten million children every day (Whitebook et al., 2018). During the COVID-19 pandemic early childhood educators were deemed “essential” by many government organizations. The early childhood care and education (ECCE) workforce serves children from birth through 8 years of age through several early learning program options for families: child care centers that can be for profit or non-profit, Early Head Start and Head Start programs that are funded through child care grants and federal government supports, as well as licensed home child care programs and unlicensed-registered ministries (Scott et al., 2017). While some early learning programs stayed open or even increased enrollment due to school age children enrolling during school closures, others had marked decreases in enrollment and/or closures. New federal, state and local guidelines dictated how early learning programs were able to operate to include children who could attend (i.e., children of essential workers only), and how many children were able to be in the program (Centers for Disease Control & Prevention, 2020). However, the changing requirements, enrollment and desire to continue educating young children (Aziegbe & Taylor-Cook, 2020) put stress not only on programs but on early childhood educators themselves.

The wellbeing of caregivers/teachers is particularly critical, not only for their own sake, but also for the wellbeing of the children they serve. “Emerging research has pointed to factors—particularly a secure attachment between child and caregiver and the emotional and mental well-being of the caregiver—that are important components of beneficial care”. (National Research Council, 2011, p. 85). Teacher–child relationship in pre-school was found to be associated with skills in first grade even after controlling for individual child’s sociodemographic variables (Hamre & Pianta, 2004). Rimm-Kaufman and Hamre (2010) argued that the quality of teachers’ interaction with children results from an interaction between teachers’ professional experience and psychological attributes. Stressed teachers suffer from depletion and are often less able to attend emotionally to children (Zinsser et al., 2013). Kwon et al. (2019) found that the psychological distress (i.e., depressive symptoms) in Early Head Start teachers was directly associated with their reports of children’s behavioral problems as well as the quality of emotional supports they were able to provide to the children. The study also speculated that the children react and learn from teachers’ negative moods with resultant behavioral issues and poor self-regulation. In a study by Hamre and Pianta (2004) of over 1,000 early childhood caregivers, those who reported having more depressive symptoms were less sensitive and more withdrawn in their interactions with children, compared to caregivers who reported fewer depressive symptoms. On the contrary, increasing early childhood educators’ emotional availability, thus allowing child-centered interactions, promotes child socio-emotional and cognitive development (Shirvanian & Michael, 2017). A review of teacher interactions with young children concluded that not just structural supports, but sensitive and responsive interactions are linked to positive child outcomes such as social skills, language development and early literacy (Norris & Horm, 2015). In a massive review about transforming the ECCE workforce, the Institute of Medicine and National Research Council (2015) concluded that young children flourish in early learning settings where there are secure and positive relationships with early childhood educators who are capable of responding to a child’s individual needs.

The increase in the need for high quality early learning programs during the pandemic has highlighted not just child well-being, but equally important, the well-being of early childhood educators who are often challenged emotionally and experience considerable stress (Yoon, 2002; Zinsser et al., 2016). Educators are impacted by a myriad of internal and external pressures which influence the day-to-day demands and increase the psychological and workload effects (Kelly & Berthelsen, 1995). In one study involving semi-structured interviews, directors showed an increase in awareness in the rise of mental stress reported by ECCE staff but were uncertain as to how to handle these mental health stressors (Logan et al., 2020). Early childhood educators often reported being overly committed to the children in their care at the cost to their own emotional well-being. This was especially true for early childhood educators teaching children in infancy and toddlerhood, and for those caring for children with traumatic life events (Kwon et al., 2021). Hall-Kenyon et al. (2014) developed a review of literature identifying only 8 articles attending to the stress of early childhood educators or, more specifically, preschool educators’ well-being. One of the key points drawn from the review was that additional research is needed (Cumming, 2016) and that research “should focus on a wider view of teacher well-being in order to support them in their work with young children” (Hall-Kenyon et al., 2014, p. 161). However, the authors also noted that the articles were fragmented and narrow in scope on issues related to such things as financial stability, emotional and physical health. It is likely that with the crisis of a global pandemic, early childhood educators’ experiences of mental and physical stress were amplified.

Financial well-being is also an important factor, not only for the early childhood educators, but also for the children for whom they care. King et al. (2015) found that when educators reported experiencing greater financial well-being—defined as wages, and perceptions of the ability to meet expenses—they seemed to demonstrate greater emotional availability in interactions with children. Early childhood educators with higher pay and experience had higher motivation to work in ECCE settings and reported less stress (Jeon et al., 2019). Yet, financial resources for early childhood educators, who often work long hours for poor pay, are limited. During the COVID-19 global pandemic early childhood educators faced an increasing shortfall for compensation due to lack of, or low, enrollment. Compensation is frequently linked with educators’ well-being (Cumming, 2016)—low compensation increases burnout and psychological distress (Al-Adwan & Al-Khayat, 2016). Teaching in early learning programs is one of the lowest-paid occupations in the United States. In 2017, median wages for early childhood educators ranged from $10.72 per hour (or $22,290 full-time per year) to $13.94 per hour (or $28,990 full-time per year) (McLean et al., 2019).

This study sought to measure early childhood educators’ personal physical, emotional and financial stress impact related to the onset of the COVID-19 global pandemic in Indiana. More research on early childhood educator stress and well-being is needed to fill a gap in the literature, especially as a national pandemic has placed unavoidable amounts of new stressors on the ECCE workforce.

## Methods

We developed an anonymous, self-administered online survey using validated measurement tools. The survey was advertised through email and social media sites geared toward early childhood educators in Indiana asking for volunteers to take the survey. This study was deemed as an exempt study by the Institutional Review Board because it was voluntary, anonymous and had minimal risk. Notices were posted from June-mid-August, 2020. The governor of Indiana enacted a “Stay-at-Home” order effective March 25, 2020—respondents were asked about each set of items before the governor’s order and then after the shutdown order.

### Sample

145 respondents completed at least the first section of the survey. The respondents were from 94 different Indiana zip codes. The mean age of the respondents was 46.8 years (median = 47); 98% were female; 16% were black, non-Hispanic, 72.4% White, non-Hispanic, 8.6% Hispanic and 2.1% Asian. 23.6% of respondents were employees, 30.6% were directors and 45.8% were owners. 23.6% worked in Ministry childcare, 38.9% in family / home, 31.9% in center-based, 2.8% Head Start and 2.1% were classified as exempt.

### Measures

Physical health was measured using a single item, “In general, how would you rate your overall health”? with 5 response categories ranging from excellent to poor.

Emotional stress was measured in two ways. The first used the Kessler 6 item mental health index (Prochaska et al., 2012). Respondents were asked “how often did you feel…” Items included sad, restless or fidgety, worthless, nervous, everything was an effort and hopeless. There were 5 response categories ranging from all to none. The second emotional stress measure asked for a series of yes/no to “Did worry or stress cause…”. Items included trouble with sleep, poor appetite or overeating, headaches or stomachaches, alcohol or drug use, difficulty controlling temper, and worsening chronic condition.

Financial stress was measured in two ways—both adapted from the Consumer Financial Protection Bureau Financial Wellbeing Scale. The first item asked whether they had money left over at the end of the month and the second asked whether they had concern that their money wouldn’t last. Response categories were always, often, sometimes, rarely or never.

Financial stress was also measured using 6 yes or no response items from the Kaiser Family Foundation’s (KFF) Health Tracking Poll (Kirzinger et al., 2020). Response items included whether they had: fallen behind on rent or mortgage, problems paying for food, problems paying for utilities, fallen behind on credit cards or bills, problems affording health insurance and problems affording prescription medications.

### Analyses

Items with 5 response categories were recoded from 1 to 5 with 5 being the highest response and 1 being the lowest response. Items with yes/no responses were recoded as 1 or 0 respectively. Paired t-tests were then used to test whether there was a significant difference between before or after COVID-19. Chi-square was used to test the difference in categorial variables. For the Kessler 6, the items were then converted to an index and individual’s scores were categorized as moderate or severe mental stress based on a cutoff of 13. All analyses were performed using SPSS v22.

## Results

### Physical Health

**Fig. 1:**
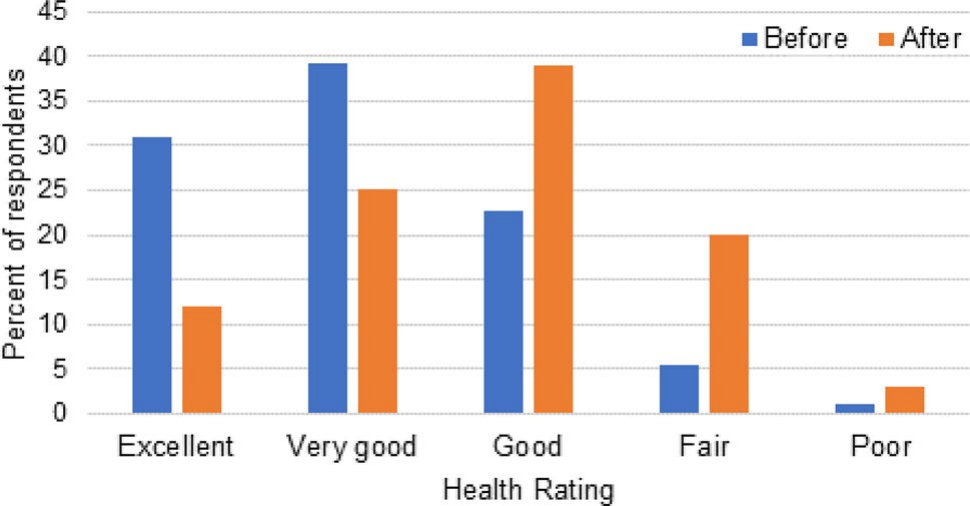
Self-reported health rating before and after Indiana’s COVID-19 stay-at-home order

General health rating was rated as very good or excellent in 70% of the respondents prior to the pandemic, whereas only 37% rated their health as very good or excellent during the shutdown period. Those rating their health as only fair or poor increased 3.5-fold. (Fig. 1).

### Emotional Stress

On individual items of the Kessler 6 Scale of Mental Stress, there were statistically significant increases in sadness, being restless or fidgety, feelings of worthlessness, nervousness, feeling everything was an effort and hopeless between before and after the COVID-19 Stay at Home order. Between 1/8 and 1/13 early childhood educators felt sad, worthless or hopeless most or all of the time. Even more (1/3 to 1/4) early childhood educators endorsed feeling restless, nervous or that everything was an effort most or all of the time (Table 1). The Kessler 6 Index (Prochaska et al., 2012) can be further analyzed to determine whether an individual has moderate or severe stress. Even before COVID-19, a third (32.8%) of early childhood educators reported feeling moderate personal stress and 4% had severe stress. Those portions rose significantly (p < 0.001) after COVID-19 with 63% reporting either moderate (46%) or severe personal stress (17.5%).

**Table 1:** Kessler 6 stress index—percentage reporting “How often did you feel…”? for each item before and after the covid-19 stay-at-home order

Item	Sad		Restless fidgety		Worthless		Nervous		Everything an effort		Hopeless	
Response	Before	After	Before	After	Before	After	Before	After	Before	After	Before	After
All	0.7	1.4	2.8	8.6	0	2.3	2.8	8.6	2.1	5.8	0	4.2
Most	2.8	6.3	2.8	15.8	1.4	6.5	4.9	19.3	5.5	15.8	2.9	8.5
Some	7.6	30.3	19.7	34.5	3.5	13	18.9	35	9	23	7.2	23.9
A little	26.4	19	28.2	25.2	19	21.7	37.1	25.7	30.3	23	20.1	21.1
None	62.5	43	46.5	15.8	76.1	56.5	36.4	11.4	49.7	32.4	69.8	42.3

**Fig. 2:**
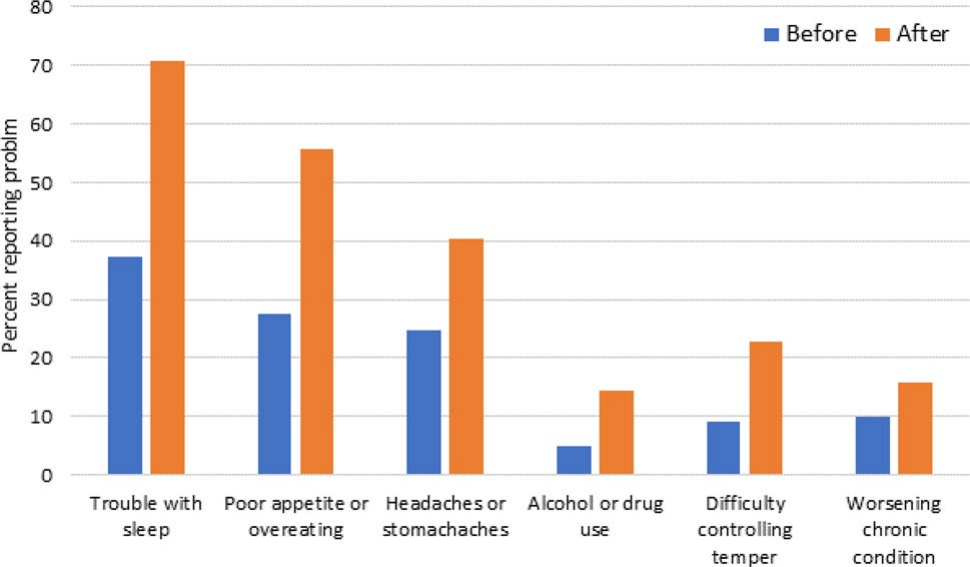
Psychosomatic symptoms before and after the pandemic stay-at-home order—response to “Did worry or stress cause…”?

Emotional Stress was also measured by changes in psychosomatic symptoms (Kirzinger et al., 2020). All these symptoms significantly increased with many symptoms doubling (sleep, poor appetite or overeating, headaches or stomachaches) or tripling (alcohol or drug use and difficulty controlling temper) in frequency (Fig. 2).

### Financial Stress

Items adapted from the CFPB financial well-being scale showed that a large portion of early childhood educators (1 out of 5) rarely or never had money left over at the end of the month even before COVID-19. That portion more than doubled with nearly half reporting they rarely or never had money left at the end of the month after COVID-19. 33% of early childhood educators always or often worried their money wouldn’t last prior to COVID-19 while 50% reported always or often having this concern after COVID-19. (Table 2).

**Table 2:** Portion of early childhood educators reporting how often they have money left over at the end of the month and concern the money wouldn’t last

Response	Money left over		Concern money wouldn’t last	
	Before (%)	After (%)	Before (%)	After (%)
Always	30.1	12.3	13.2	24.6
Often	21.7	14.5	20	25.4
Sometimes	29.4	27.5	29.9	27.5
Rarely	15.4	32.6	23.6	10.9
Never	3.5	12	13.2	11.8

**Fig. 3:**
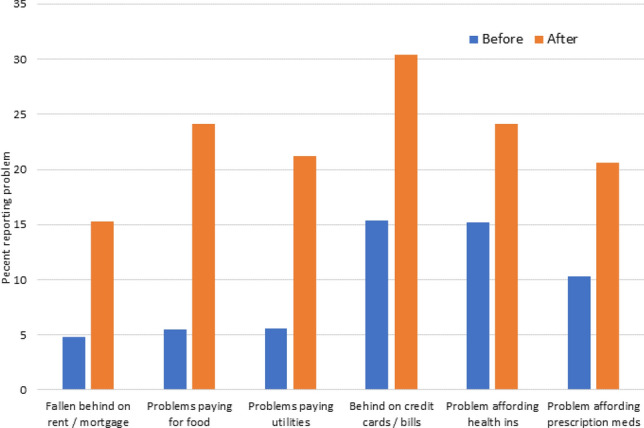
Personal financial stressors before and after the pandemic stay-at-home order

The early childhood educators also reported doubling to quadrupling on most of the other measures of financial stress from the KFF Health Tracking poll (Kirzinger et al., 2020). The largest increases were in problems paying for food, paying for utilities and falling behind on rent/mortgage. (Fig. 3).

## Discussion

Recent research and raw unpublished data during the COVID-19 pandemic primarily reflects the social and emotional health of young children and children in K-12 educational settings. Research shows that early childhood educators create learning environments that greatly influence the experiences of young children (Burchinal et al., 2002; Hamre & Pianta, 2001). However, there is far less documented research on early childhood educators’ well-being, especially during a global health pandemic such as COVID-19. Studies in the early 2000s and emerging research, have begun to focus on the early childhood educators’ wellbeing and psychological stress (both personal and workplace) with the concomitant emotional responses that impact children’s learning, teacher child-interactions and overall social and emotional regulation (Jennings & Greenburg, 2009; Jeon et al., 2018; Early et al., 2006; Whitaker et al., 2015;). Our study further supports the findings of Kwon et al. (2020) that the majority of the ECCE workforce have disparities in psychological and physical well-being, and also found that the COVID-19 pandemic has had a huge impact and even widened these disparities.

Although, the physical health impacts on early childhood educators have been described prior to the pandemic, including increased exposure to various infectious diseases (e.g., viral, respiratory and skin) and other occupational hazards such as musculoskeletal pain (Bradley, 2003; King et al., 2006), the respondents to our survey rated their overall health as similar to those in a national sample (National Center for Health Statistics, 2015). However, the early childhood educators who participated in our study reported markedly worse mental health stress. Even before the pandemic early childhood educators reported more moderate (32.8 vs 18.6%) and serious distress (3.9 vs 3.4) than a national sample. The Centers for Disease Control and Prevention, in nationally conducted surveys using the Kessler-6 scale, documented rates of “psychological distress” of between 3.2 and 4.0% (Reeves et al., 2011). Early childhood educators are reporting a high level of overall psychological symptoms compared to individuals in the community and the portion of early childhood educators experiencing moderate of serious distress nearly doubled during the pandemic. In 2019, over 2 million paid early childhood educators represented a highly vulnerable workforce (Linnan et al., 2017; Whitebook et al., 2018), with overrepresentation of women, people of color, and people of low socioeconomic status who experience inequities related to well-being, financial and psychological stress (Whitebook et al., 2004, 2018). Whitaker et al. (2015) reported that external factors such as family caregiving and personal financial stress—factors likely highly impacted by the pandemic—increase educator stress and influence teacher–child interactions.

Physical and behavioral symptoms of stress were 2–3 times higher during COVID-19 as compared to before. Perhaps the most troubling, from an early childhood perspective, is reports of difficulty controlling their temper with almost one out of ten reporting difficulty before and nearly one out of four early childhood educators reporting difficulties during COVID-19. Early childhood educators’ mental health (depressive) symptoms are known to influence the quality of their teaching practice and their interactions with children (Jeon et al., 2014; Sandilos et al., 2015). At times (such as during a pandemic) when children may also be experiencing stress and behavior issues providing a safe, structured nurturing environment is even more important, so the health and mental health of their providers is paramount. Early childhood educators reported markedly higher rates (ranging from 24 to 77% higher) in each item than Kaiser Family Foundation national sample taken at a similar time (trouble with sleep 71 vs 40%; poor or overeating 56 vs 33%, headaches or stomachaches 40 vs 18% and worsening chronic conditions16 vs 9%) during COVID-19. They reported similar responses to increases in alcohol or drug use (14.3 vs 13% respectively) (Kirzinger, 2020).

Early childhood educators also reported financial concerns exacerbated by the pandemic. Reports of financial concerns were 2 to 4 times higher during COVID-19. Although early childhood educators reported similar rates of falling behind on their rent or mortgage as compared to a national sample (15.3 vs 15%, respectively) they had increases of 30–70% for financial concerns in paying for food (24 vs 14%), paying for utilities (21 vs 16%) and paying for credit card bills (30 vs 21%). Most striking was that, even before COVID-19, early childhood educators reported twice as many problems affording health insurance and affording prescription medicines than the national sample did at the time of COVID-19. After COVID-19, responses were as much as 4 times higher than the national sample with one in four to one in five early childhood educators in our study having problems affording health insurance and paying for prescription medications. This is reflective of reports demonstrating a lack of benefits available to early childhood educators (Whitebook et al., 2016). Otten et al. (2019) reported that early childhood educators earn lower wages, experience poor mental well-being, and have higher rates of food insecurity. The majority of early childhood educators worked full time, had a college degree and at least ten years of teaching experience. Of those interviewed 23 percent reported using public assistance due to low wages. Despite the crucial nature of the ECCE workforce (especially during COVID-19), they remain among the country’s lowest paid workers, and rarely receive health insurance, retirement benefits or other job-based benefits (Gould, 2015). Half of all early childhood educators are enrolled in at least one public support program, such as the Supplemental Nutrition Assistance Program or public health insurance i.e., Medicaid (Lessard et al., 2020). Over 14% of early childhood educators live in families with incomes below the official poverty line, twice as many as workers in other occupations (6.7%) (Gould, 2015). Three times as many workers in other occupations receive employer-sponsored health insurance compared to ECCE workers (49.9 vs 15%, respectively) (Gould, 2015). These financial stressors, in particular, likely contributed to the increased stress seen in our study during this national health pandemic. Interestingly, in the Whitaker et al. (2015) study, many teachers remarked on the impact of financial hardships, but it was not significantly associated with the quality of relationships they had with children in their care. Regardless, multiple studies have shown the association of economic deprivation (food, housing, clothing) and stress associated with low incomes to have large impacts on individual’s health, rates of chronic diseases, and a shortened life expectancy (Evans & English, 2002; Kanjilal et al., 2006; Singh & Siahpush, 2006).

The COVID-19 pandemic has impacted all sectors of the workforce, in particular those considered “essential”. A large portion of individuals working in healthcare are parents of young children and found an increased reliance on early childhood education programs. In a recent study, Nagasawa and Tarrant (2020) reported that the calls to “reopen the economy” intensified the attention given to the early childhood and care workforce, recognizing that child care is not just a parent responsibility but a societal one. Further stating that the United States must understand that early care and education is a foundational part of the economy that impacts both early childhood well-being and psychological stress seen in the workplace.

Our study has several limitations. First, we utilized a cross-sectional design with all the data collected at the same time via a survey. Participants were asked to respond to items before the onset of the COVID-19 stay-at-home order and may not have recalled it accurately. It is not known whether re-call bias would systematically increase or decrease prior ratings of physical health, emotional stress and financial stress. Using survey or self-reported data does not allow triangulation or validation of our findings through multiple data sources. Our data, however, are consistent with other reports of stress in early childhood educators. Additionally, other variables, such as educational background, years of experience, teachers of specific age groups of children (infant/toddlers, preschoolers), and work status (full-time work, unemployed, part-time work etc.) have been shown to be related to teacher stress and were unmeasured in our sample. It would be interesting to sample only infant and toddler educators in the aftermath of the COVID-19 pandemic, as early childhood educators may have different needs, as well as physical and emotional risks, depending on both the age group they work with and their level of education (Kwon et al., 2020). Our sample size was small but with good geographic distribution within our state. A large longitudinal sample to monitor outcomes for early childhood educators is needed—even if COVID-19 is controlled, the baseline data reported are concerning enough that national attention should focus on early childhood educators as vulnerable, essential providers dealing with multiple stressors.

## Conclusion

Before the pandemic, 43% of children 0–5 years of age worldwide were estimated to be at risk of not achieving their optimal developmental potential (Roberton et al., 2020). This percentage reflects the importance of early childhood educators’ health and well-being, a largely neglected issue in the early childhood and care workforce. Hall-Kenyon et al. (2014) stated “efforts to improve education for young children should not only emphasize what teachers do when teaching but also who they are and how they are affected by the doing”. This current study contributes to a growing literature on early childhood educators’ physical, psychological and financial stress and illuminates how a global pandemic, such as COVID-19, increases those disparities in this educator group. Deemed “essential workers” during the COVID-19 crisis, our study further supports the growing call (Linnan et al., 2017) for administrators, policy makers and stakeholders to provide improved supports for the psychological, physical and financial well-being of the ECCE workforce that is consistently overworked, underpaid, and yet, invaluable. Knowing the varying dimensions and time period of the COVID-19 global pandemic, the time is now for educators, policymakers and advocates to share this knowledge and gain better insight into the risk factors prior to the pandemic and those exacerbated by the pandemic that impact the overall physical, psychological and financial well-being of this vulnerable essential workforce.

## References

[CR1] Al-Adwan FE, Al-Khayat MM (2016). Psychological burnout in early childhood teachers: Levels and reasons. International Education Studies.

[CR2] Aziegbe E, Cook T (2020). Rising to the challenge. Child care programs fill emotional gaps during the pandemic. Exchange the Early Childhood Leaders Magazine.

[CR3] Bradley RH (2003). Child care and common communicable illnesses in children aged 37 to 54 months. Archives of Pediatrics & Adolescent Medicine.

[CR4] Burchinal MR, Cryer D, Clifford RM, Howes C (2002). Caregiver training and classroom quality in child care centers. Applied Developmental Science.

[CR5] Centers for Disease Control and Prevention. (2020). Interim guidance for administrators of U.S. K-12 schools and child care programs plan, prepare, and respond to coronavirus disease 2019 (COVID-19). https://www.cdc.gov/coronavirus/2019/-ncov/community/schools-childcare/guidance-for-schools-html

[CR6] Cumming T (2016). Early childhood educators’ well-being: An updated review of literature. Early Childhood Education Journal.

[CR7] Early DM, Bryant DM, Pianta RC, Clifford RM, Burchinal MR, Ritchie S, Howes C, Barbarin O (2006). Are teachers’ education, major, and credentials related to classroom quality and children’s academic gains in pre-kindergarten?. Early Childhood Research Quarterly.

[CR8] Evans GW, English K (2002). The environment of poverty: Multiple stressor exposure, psychophysiological stress, and socioemotional adjustment. Child Development.

[CR9] Gould, E. (2015). Child care workers aren’t paid enough to make ends meet.* Economic Policy Institute, 405,* 1–19. https://www.epi.org/publication/child-care-workers-arent-paid-enough-to-make-ends-meet/

[CR10] Hamre BK, Pianta RC (2001). Early teacher–child relationships and the trajectory of children’s school outcomes through eighth grade. Child Development.

[CR11] Hamre BK, Pianta RC (2004). Self-reported depression in nonfamilial caregivers: Prevalence and associations with caregiver behavior in child-care settings. Early Childhood Research Quarterly.

[CR12] Hall-Kenyon KM, Bullough RV, MacKay KL, Marshall EE (2014). Preschool teacher well-being: A review of the literature. Early Childhood Education Journal.

[CR13] Institute of Medicine [IOM] & National Research Council [NRC]. (2015).* Transforming the workforce for children birth to age 8: A unifying foundation.* The National Academies Press, Washington. 10.17226/19401 26269871

[CR14] Jennings PA, Greenberg MT (2009). The prosocial classroom: Teacher social and emotional competence in relation to student and classroom outcomes. Review of Educational Research.

[CR15] Jeon L, Buettner CK, Snyder AR (2014). Pathways from teacher depression and child-care quality to child behavioral problems. Journal of Consulting and Clinical Psychology.

[CR16] Jeon L, Buettner CK, Grant AA (2018). Early childhood teachers’ psychological well-being: Exploring potential predictors of depression, stress, and emotional exhaustion. Early Education and Development.

[CR17] Jeon HJ, Kwon KA, Walsh B, Burnham MM, Choi YJ (2019). Relations of early childhood education. Teachers’ depressive symptoms, job-related stress, and professional motivation to beliefs about children and teaching practices. Early Education and Development.

[CR18] Kanjilal S, Gregg EW, Cheng YJ, Zhang P, Nelson DE, Mensah G, Beckles GL (2006). Socioeconomic status and trends in disparities in 4 major risk factors for cardiovascular disease among US adults, 1971–2002. Archives of Internal Medicine.

[CR19] Kelly AL, Berthelsen DC (1995). Preschool teachers’ experiences of stress. Teaching and Teacher Education.

[CR20] King PM, Gratz R, Kleiner K (2006). Ergonomic recommendations and their impact on child care workers’ health. Work.

[CR21] King EK, Johnson AV, Cassidy DJ, Wang YC, Lower JK, Kintner-Duffy VL (2015). Preschool teachers’ financial well-being and work time supports: Associations with children’s emotional expressions and behaviors in classrooms. Early Childhood Education Journal.

[CR22] Kirzinger, A., Hamel, L., Munana, C., Kearney, A., & Brodie, M. (2020). *KFF health tracking poll—late April 2020: Coronavirus, social distancing, and contact tracing—economic and mental health impacts*. KFF. https://www.kff.org/report-section/kff-health-tracking-poll-late-april-2020-economic-and-mental-health-impacts-of-coronavirus/?utm_campaign=KFF-2020-polling-surveys&utm_source=hs_email&utm_medium=email&utm_content=86917437&_hsenc=p2ANqtz--89fbrxjVE6vIGvlQ_77kiaSBhSaYb_RinGOnY41WkARf-1FgKfiDDxHD1TvUK3EZ-PsGcFKOqPZOZumvA1tV1GJVkCA&_hsmi=86917437

[CR23] Kwon KA, Jeon S, Jeon L (2019). The role of teachers’ depressive symptoms in classroom quality and children’s developmental outcomes in early head start programs. Learning and Individual Differences.

[CR24] Kwon, K., Ford, T. G., Salvatore, A. L. Randall, K., Jeon, L., Malek-Lasater, A., Ellis, N., Kile, M. S., Horm, D. M., Kim, S. G., & Han, M. (2020). Neglected elements of a high-quality early childhood workforce: Whole teacher well-being and working conditions.* Early Childhood Education Journal*, 1–12. 10.1007/s10643-020-01124-7

[CR25] Kwon, K., Horm, D. M., & Amirault, C. (2021). Early childhood teachers’ well-being: What we know and why we should care.* ZERO to THREE Journal, 41*(3), 35–44. https://www.zerotothree.org/resources/3912-early-childhood-teachers-well-being-what-we-know-and-why-we-should-care

[CR26] Lessard LM, Wilkins K, Rose-Malm J, Mazzocchi MC (2020). The health status of the early care and education workforce in the USA: A scoping review of the evidence and current practice. Public Health Reviews.

[CR27] Linnan L, Arandia G, Bateman LA, Vaughn A, Smith N, Ward D (2017). The health and working conditions of women employed in child care. International Journal of Environmental Research and Public Health.

[CR28] Logan H, Cumming T, Wong S (2020). Sustaining the work-related wellbeing of early childhood educators: Perspectives from key stakeholders in early childhood organisations. International Journal of Early Childhood.

[CR29] McLean, C., Whitebook, M., & Roh, E. (2019). *From unlivable wages to just pay for early educators*. Center for the Study of Child Care Employment, University of California, Berkley. https://cscce.berkeley.edu/from-unlivable-wages-to-just-pay-for-early-educators/

[CR30] Nagasawa, M., & Tarrant, K. (2020). *Who will care for the early care and education workforce? COVID-19 and the need to support early childhood educators’ emotional well-being*. New York Early Childhood Professional Development Institute, CUNY. https://educate.bankstreet.edu/sc/1

[CR31] National Center for Health Statistics. (2015). *Survey Description, National Health Interview Survey*. Hyattsville, Maryland. 2016. https://ftp.cdc.gov/pub/Health_Statistics/NCHS/Dataset_Documentation/NHIS/2015/srvydesc.pdf

[CR32] National Research Council, Institute of Medicine, Board on Children. (2011). *The early childhood care and education Workforce: Challenges and opportunities: A workshop report*. Challenges and Opportunities: A Workshop Report|The National Academies Press. https://www.nap.edu/catalog/13238/the-early-childhood-care-and-education-workforce-challenges-and-opportunities 24624473

[CR33] Norris DJ, Horm DM (2015). Research in review: Teacher interactions with infants and toddlers. Young Children.

[CR34] Otten JJ, Bradford VA, Stover B, Hill HD, Osborne C, Getts K, Seixas N (2019). The culture of health in early care and education: Workers’ wages, health, and job characteristics. Health Affairs.

[CR35] Prochaska JJ, Sung HY, Max W, Shi Y, Ong M (2012). Validity study of the K6 scale as a measure of moderate mental distress based on mental health treatment need and utilization. International Journal of Methods in Psychiatric Research.

[CR36] Reeves, W. C., Pratt, L. A., Thompson, W., Ahluwalia, I. B., Dhingra, S. S., McKnight-Eily, L. R., & Gould, D. (2011). *Mental illness surveillance among adults in the United States. Morbidity and Mortality Weekly Report (MMWR)*. Centers for Disease Control and Prevention. https://www.cdc.gov/mmwr/preview/mmwrhtml/su6003a1.htm 21881550

[CR37] Rimm-Kaufman SE, Hamre BK (2010). The role of psychological and developmental science in efforts to improve teacher quality. Teachers College Record.

[CR38] Roberton T, Carter ED, Chou VB, Stegmuller AR, Jackson BD, Tam Y, Walker N (2020). Early estimates of the indirect effects of the COVID-19 pandemic on maternal and child mortality in low-income and middle-income countries: a modelling study. The Lancet Global Health.

[CR39] Sandilos LE, Cycyk LM, Hammer CS, Sawyer BE, Lopéz L, Blair C (2015). Depression, control, and climate: An examination of factors impacting teaching quality in preschool classrooms. Early Education and Development.

[CR40] Scott K, Looby AA, Hipp JS, Frost N (2017). Applying an equity lens to the child care setting. Journal of Law Medicine & Ethics.

[CR41] Shirvanian, N., & Michael, T. (2017). Implementation of attachment theory into early childhood settings. *International Education Journal: Comparative Perspectives, 16*(2), 97–115. https://openjournals.library.sydney.edu.au/index.php/IEJ/article/view/10978.

[CR42] Singh GK, Siahpush M (2006). Widening socioeconomic inequalities in US life expectancy, 1980–2000. International Journal of Epidemiology.

[CR43] Whitaker RC, Dearth-Wesley T, Gooze RA (2015). Workplace stress and the quality of teacher–children relationships in head start. Early Childhood Research Quarterly.

[CR44] Whitebook, M., Phillips, D., Bellm, D., Crowell, N., Almaraz, M., & Jo, J. Y. (2004). *Two years in early care and education: A community portrait of quality and workforce stability*. Berkeley, CA: Center for the Study of Child Care Employment, University of California at Berkeley. https://cscce.berkeley.edu/wp-content/uploads/2004/twoyears_final.pdf

[CR45] Whitebook, M., King, E., Philipp, G., & Sakai, L. (2016). *Teachers’ voices: Work environment conditions that impact teacher practice and program quality*. Center for the Study of Child Care Employment, University of California at Berkeley. https://cscce.berkeley.edu/wp-content/uploads/2016/2016-Alameda-SEQUAL-Report-FINAL-for-Dissemination-v2.pdf

[CR46] Whitebook, M., McLean, C., Austin, L.J.E., & Edwards, B. (2018). *Early childhood workforce index—2018*. Berkeley, CA: Center for the Study of Child Care Employment, University of California, Berkeley. http://cscce.berkeley.edu/topic/earlychildhood-workforce-index/2018/

[CR47] Yoon JS (2002). Teacher characteristics as predictors of teacher-student relationships: Stress, negative affect, and self-efficacy. Social Behavior and Personality an International Journal.

[CR48] Zinsser KM, Bailey CS, Curby TW, Denham SA, Bassett HH (2013). Exploring the predictable classroom: Preschool teacher stress, emotional supportiveness, and students social-emotional behavior in private and Head Start classrooms. Dialog.

[CR49] Zinsser KM, Christensen CG, Torres L (2016). She's supporting them; who's supporting her? Preschool center-level social-emotional supports and teacher well-being. Journal of School Psychology.

